# Improvement in Microbiota Recovery Using Cas-9 Digestion of Mānuka Plastid and Mitochondrial DNA

**DOI:** 10.1007/s00248-024-02436-6

**Published:** 2024-10-09

**Authors:** J. L. Larrouy, H. J. Ridgway, M. K. Dhami, E. E. Jones

**Affiliations:** 1 Department of Pest-Management and Conservation, Faculty of Agriculture and Life Sciences, Lincoln University, Lincoln Christchurch, 7647 New Zealand; 2 Biocontrol & Molecular Ecology, Manaaki Whenua Landcare Research, Lincoln, 7608 New Zealand; 3 The New Zealand Institute for Plant and Food Research Limited, Lincoln, 7608 New Zealand

**Keywords:** Plant microbiome, 16S rRNA gene amplicon sequencing, Cas-16S-seq, Host contamination, Microbiota profiling

## Abstract

Understanding host-microbe interactions *in planta* is an expanding area of research. Amplicon sequencing of the 16S rRNA gene is a powerful and common method to study bacterial communities associated with plants. However, the co-amplification of mitochondrial and plastid 16S rRNA genes by universal primers impairs the sensitivity and performance of 16S rRNA sequencing. In 2020, a new method, Cas-16S-seq, was reported in the literature to remove host contamination for profiling the microbiota in rice, a well-studied domestic plant, by engineering RNA-programmable Cas9 nuclease in 16S rRNA sequencing. For the first time, we tested the efficiency and applicability of the Cas-16S-seq method on foliage, flowers, and seed of a non-domesticated wild plant for which there is limited genomic information, *Leptospermum scoparium* (mānuka). Our study demonstrated the efficiency of the Cas-16S-seq method for *L. scoparium* in removing host contamination in V4-16S amplicons. An increase of 46% in bacterial sequences was found using six guide RNAs (gRNAs), three gRNAs targeting the mitochondrial sequence, and three gRNAs targeting the chloroplast sequence of *L. scoparium* in the same reaction. An increase of 72% in bacterial sequences was obtained by targeting the mitochondrial and chloroplast sequences of *L. scoparium* in the same sample at two different steps of the library preparation (DNA and 1st step PCR). The number of OTUs (operational taxonomic units) retrieved from soil samples was consistent when using the different methods (Cas-16S-seq and 16S-seq) indicating that the Cas-16S-seq implemented for *L. scoparium* did not introduce bias to microbiota profiling. Our findings provide a valuable tool for future studies investigating the bacterial microbiota of *L. scoparium* in addition to evaluating an important tool in the plant microbiota research on other non-domesticated wild species.

**Supplementary Information:**

The online version contains supplementary material available at 10.1007/s00248-024-02436-6.

## Introduction

Understanding the impact of plant-associated microbes on their host and other microbes, particularly how they can benefit or be engineered to benefit their host is highly valuable to mitigate imminent threats such as climate change or pathogen incursions [1, 2]. Thus, the number of plant microbiome studies has drastically increased in recent years [3].

Research on bacterial communities associated with plants broadly uses the 16S ribosomal RNA (rRNA) gene amplicon sequencing method. This method consists of extracting DNA from plant samples allowing the concurrent extraction of microbial DNA associated with the plant to be studied. From this pooled DNA, a specific region of the bacterial 16S rRNA gene is amplified using PCR and conserved primers to obtain amplicons of this region from all the different bacterial species present in the sample. The resulting amplicons are sequenced, allowing the bacterial community to be characterised using bioinformatic tools.

However, universal primers for 16S rRNA gene amplicon sequencing also amplify the eukaryotic mitochondria and plastid 16S rRNA genes in plants, which are derived from prokaryotic ancestors [4]. These organelles are generally more abundant in plant tissue than microbes. Thus, the amount of plant DNA will be higher than microbial DNA, affecting the amplification efficiency, due to the co/amplification of plant and bacterial DNA. Indeed, host contamination (plastid and mitochondrial sequences) can represent up to 95% of all reads in 16S rRNA amplicon sequencing from plant samples [5–7]. Such a high degree of host contamination impairs plant microbiota studies.

Several methods to overcome host contamination in 16S rRNA sequencing such as discriminating primers (designed to selectively bind to bacterial DNA), PCR clamps (modified oligonucleotides binding to plant DNA during PCR to prevent amplification), and blocking oligos (nested PCR to ‘poison’ organellar amplicons) have been used for several years [6, 8, 9]. However, their efficiency is highly variable across plant species, and they can introduce bias in microbiota profiling [10].

A method using the Cas9 enzyme and specific single-guide RNA (sgRNA) was recently developed to decrease host contamination by exclusively cleaving host 16S rRNA gene amplicons and subsequently enriching bacterial sequences during the preparation of 16S-seq amplicon libraries [11]. Using this Cas-16S-seq method, the authors reduced, on average, rice 16S rRNA gene sequences from 63.2 to 2.9% in root samples and from 99.4 to 11.6% in phyllosphere samples. CRISPR (clustered regularly interspaced short palindromic repeat)-Cas is an adaptive immune system in bacteria and archaea and is extensively used as a tool to precisely manipulate DNA. The Cas enzyme modifies a template by creating a double-strand break at a specific site, guided by RNA. The sgRNA consists of two parts, CRISPR-RNA (crRNA) complementary to the target DNA, and trans-activating crRNA (tracrRNA) serving as a binding scaffold for the Cas nuclease. Using a sgRNA complementary to a target region, a Cas9 enzyme from *Streptococcus pyogenes*, can be directed to cleave the target DNA bearing an upstream protospacer-adjacent motif (PAM). For Cas9, the double-strand break occurs three bases upstream of the PAM. The enzyme can therefore easily be reprogrammed to cleave any DNA sequence by replacing the 20-nucleotide crRNA at the 5′-end of gRNA.

This method worked well in rice but remained untested in other species, especially wild, non-domesticated species for which there is limited genetic information. In this study, we assessed the broader applicability of the Cas-16S-seq method to *Leptospermum scoparium* (mānuka), to investigate bacterial microbiota recovery from a wild, non-crop species. We designed specific gRNAs that discriminate *L. scoparium* 16S rRNA genes from bacterial and archaeal 16S rRNA genes and optimised the Cas-16S-seq method to find optimal gRNAs and cleavage protocol. We then compared Cas-16S-seq to standard 16S rRNA amplicon sequencing (16S-seq) using multiple *L. scoparium* samples (flower, leaf, and seed), representing three genotypes. *L. scoparium* is an economically important native shrub in New Zealand and Australia, due to the honey produced from its nectar. There is particular interest in the microbiome of *L. scoparium* flowers to enhance honey quality. In addition, *L. scoparium* is part of the widely distributed Myrtaceae family that contains over 5000 species in tropical and warm-temperate regions, thus global application of this method to wild Myrtaceae can be anticipated.

## Materials and Methods

### Design of gRNAs Targeting *L. scoparium* Chloroplast and Mitochondrial Sequences and In Vitro Cleavage Testing of the gRNAs

A total of 24 flowers from three ‘Crimson Glory’ *L. scoparium* mature plants located at the Plant & Food Research Ltd, Palmerston North site (− 40.377089, 175.615581), New Zealand, were collected in October 2019. A sample corresponded to a single flower. The tubes containing a single flower were snap-frozen in liquid nitrogen and stored at − 80 °C prior to DNA extraction. DNA extraction was performed using the Nucleospin 96 Plant II kit (Marchery-Nagel, Germany) following the manufacturer’s instructions but including small modifications. The plant samples were snap frozen in liquid nitrogen before being bead beaten until finely powdered; 3 times for 30 s at maximum speed (2010 Geno/Grinder, SPEXSamplePrepP). For the cell lysing step, buffer PL2 and PL3 were added before a 60-min incubation at 65 °C. *L. scoparium* chloroplast and mitochondrial 16S rRNA gene sequences were obtained by amplifying the V4 region of the 16S rRNA gene from flower DNA, using primers 515f (5′GTG YCA GCM GCC GCG GTA A-3′)—806r (5′- GGA CTA CNV GGG TWT CTA AT-3′) [12] traditionally used for bacterial community characterisation on an Illumina MiSeq sequencer with 250 × 2 paired-end mode. CRISPR-P (version 2.0) was used to extract gRNA sequences (Cas9-targetable 20 bp sequences preceding PAM, 5′-NGG-3′) from *L. scoparium* chloroplast (cp-gRNA) and mitochondrial (mt-gRNA) sequences. The established bioinformatics pipeline from Song and Xie [11] was used to select gRNAs that specifically target *L. scoparium* 16S rRNA genes without bacterial 16S rRNA off-targets. Briefly, 20 bp sequences upstream the 5′-NGG-3′ and 5′-NAG-3′ PAMs of prokaryotic 16S rRNA gene sequences from the Ribosomal Database Project (RDP) were extracted as RDP-rRNA dataset. Pair-wise global alignment was performed between gRNA guide sequences and RDP-rRNA dataset using the VSEARCH program as describe in Song and Xie [11]. Then, the RDP-rRNA off-targets of cp-gRNA and mt-gRNA were identified according to criteria describe in Song and Xie [11]. The total number of RDP-rRNA off-targets for each gRNA was counted (Table 1). The six gRNAs were choose based on their percentage of efficiency provided from gRNA design online tools and their lower number of off-target.

**Table 1:** gRNA targeting *Leptospermum scoparium* chloroplast and mitochondrial V4-16S rRNA gene (mt-gRNA and cp-gRNA). The table presents the gRNA ID, efficiency score calculated by CRIPSR-P tool (higher score indicates a higher cleavage performance), strand and position of gRNA on the target sequences, percentage of GC in gRNA sequence, the guide sequence, number of bacterial off-targets (NGG and NAG) for each gRNA found against 16S rRNA genes in the RDP database and sequence origin and region of 16S rRNA genes

gRNA ID	Score CRISPR-P	Strand	Position	%GC	Guide sequence (5′-3′)	Off-target NGG	Off-target NAG	16S region	Sequence origin
cp-131	0.5161	+	131	55%	ACCAAGCTGGAGTACGGTAG	0	0	V4	Chloroplast
cp-189	0.4235	+	189	45%	GAGATCGGAAAGAACACCAA	0	0	V4	Chloroplast
cp-81	0.7171	+	81	55%	AAGTCCGCCGTCAAATCCCA	0	0	V4	Chloroplast
mt-88	0.2243	+	88	40%	AAGTGAAAGTCGCCAAAAAG	2	0	V4	Mitochondrial
mt-91	0.7952	+	91	45%	TGAAAGTCGCCAAAAAGTGG	3	0	V4	Mitochondrial
mt-107	0.2235	-	107	45%	TCTGTCTCACTCAAGTGAAT	3	1	V4	Mitochondrial

A total of six gRNAs, three mt-gRNAs targeting the mitochondrial sequence of *L. scoparium*, and three cp-gRNAs targeting the chloroplast sequence of *L. scoparium* were prepared according to the manufacturer’s instructions (IDT, Australia). Each gRNA was a duplex made of a crRNA that provides the sequence specificity (guide sequences in Table 1) that is complementary to the target DNA and the tracrRNA, a universal RNA molecule that does not change for each new target sequence but must be bound to crRNA to create a functional, targeting ribonucleoprotein (RNP) with the enzyme. Each RNA oligo (Alt-R CRISPR-Cas9 crRNA and Alt-R CRISPR-Cas9 tracrRNA) was resuspended in 1X TE, pH7.5 to a stock concentration of 100 µM. The crRNA and tracrRNA oligos (100 µM) were mixed at equimolar concentration to a final duplex concentration of 10 µM. For a final volume of 10 µl, 1 µl of 100 µM Alt-R CRISPR-Cas9 crRNA, 1 µl of 100 µM Alt-R CRISPR-Cas9 tracrRNA and 8 µl of Nuclease-Free Duplex Buffer (IDT, Australia). The duplex was heated at 95 °C for 5 min and then allowed to cool at room temperature. RNP complexes were prepared according to the manufacturer’s instructions (IDT, Australia). In a final volume of 100 µl, 10 µl of 10 µM Alt-R guide RNA (crRNA:tracrRNA duplex), 1.6 µl of Alt-R S.p. Cas9 enzyme (62 µM stock), and 88.4 µl of phosphate-buffered saline (PBS) were mixed and the tube was then incubated for 10 min at room temperature. When using multiple gRNAs in the same reaction, RNP complexes were mixed at equimolar concentration, mixes are detailed in Table 2. In vitro amplicon cleavages were performed for 1 h at 37 °C in a 20 µl reaction containing 2 µl of 1 µM RNP (RNP complex used depended on the gRNA tested, e.g. 1 µl of mt-88 RNP and 1 µl of cp-81 RNP when testing mix1, in control samples, milliQ water replaces RNP complex), 50 nM of 1st step PCR amplicons (V4 region of 16S rRNA gene amplify from *L. scoparium* ‘Crimson Glory’ flower as described in the ‘Optimisation of Cas-16S-seq amplicon libraries on *L. scoparium* floral DNA’ section) and 2 µl of 10X Cas9 Nuclease Reaction Buffer (200 nM HEPES, 1 M NaCl, 50 mM MgCl2 and 1 mM EDTA, pH 6.5 at 25 °C, IDT, Australia). The reaction was treated with 2 µl of Proteinase K (20 mg/ml) at 56 °C for 10 min to release the DNA substrate from the Cas9 endonuclease. To check the cleavage efficiency of the gRNAs, 10 µl of each reaction was loaded onto a 2% agarose gel.

**Table 2:** gRNA combination targeting *Leptospermum scoparium* chloroplast and or mitochondrial V4-16S rRNA gene (mt-gRNA and cp-gRNA). The table presents the gRNA mix number, cp-gRNA and or mt-gRNA ID, target DNA origin

*gRNA combination*	*cp-gRNA*	*mt-gRNA*	Target DNA
Mix-1	cp-81	mt-88	Chloroplast and mitochondrial
Mix-2	cp-131	mt-88	Chloroplast and mitochondrial
Mix-3	cp-189	mt-88	Chloroplast and mitochondrial
Mix-4	cp-81	mt-91	Chloroplast and mitochondrial
Mix-5	cp-131	mt-91	Chloroplast and mitochondrial
Mix-6	cp-189	mt-91	Chloroplast and mitochondrial
Mix-7	cp-81	mt-107	Chloroplast and mitochondrial
Mix-8	cp-131	mt-107	Chloroplast and mitochondrial
Mix-9	cp-189	mt-107	Chloroplast and mitochondrial
Mix-10	all cp	all mt	Chloroplast and mitochondrial
Mix-11	-	mt-88/mt-91	Mitochondrial
Mix-12	-	mt-88/mt-107	Mitochondrial
Mix-13	-	mt-107/mt-91	Mitochondrial
Mix-14	cp-81/cp-131	-	Chloroplast
Mix-15	cp-81/cp-189	-	Chloroplast
Mix-16	cp-131/cp-189	-	Chloroplast

### Optimisation of Cas-16S-seq Amplicon Libraries on *L. scoparium* Floral DNA

The Cas-16S-seq method for *L. scoparium* was first optimised by characterising the optimal gRNA combination and the optimal step (during library preparation) for plant sequences cleavage on four *L. scoparium* ‘Crimson Glory’ floral samples. To confirm that gRNAs were not targeting bacterial sequences and therefore introducing bias, this method was tested on four soil samples.

The NucleoSpin 96 Plant II (Marcherey-Nagel, Germany) kit was used to extract floral DNA (2 flowers per sample) and the MN NucleoSpin Soil (Marcherey-Nagel, Germany) kit was used to extract soil DNA. The soil was sampled with a soil probe (core sample approximately 23 cm long by 1.9 cm in width) from a pasture site in Lincoln (New Zealand) and ~ 500 mg was used for DNA extraction.

To characterise the optimal gRNA combination, a total of 16 mixes with the different gRNA were tested (Table 2) and in vitro cleavage reaction was performed at DNA level. To characterise the optimal step (during library preparation) for plant sequences cleavage, gRNAs cp-189 and mt-88 were used.

In vitro DNA cleavages were performed at 37 °C for an hour in a 10 µl reaction; 2 µl of 1 µM RNP (RNP complex used depended on the gRNA tested and water was used instead of RNP complex in control condition), 30 ng of DNA substrate and 2 µl of 10X Cas9 Nuclease Reaction Buffer. After cleavage, all reactions were treated with 2 µl of Proteinase K (20 mg/ml) at 56 °C for 10 min to release the DNA substrate from the Cas9 endonuclease and then purified with SeraPure magnetic beads following the recommended Ampure magnetic bead protocol. In the first step PCR, the V4 region of the bacterial 16S rRNA gene was amplified from the cleaved DNA or uncleaved DNA (when testing the efficiency of in vitro cleavage of 1st step amplicon only) using the specific primer pairs, 515f (5′GTG YCA GCM GCC GCG GTA A-3′) – 806r (5′- GGA CTA CNV GGG TWT CTA AT-3′) fused with 3–6‐mer Ns for improved Illumina sequencing quality (forward, 5′‐ TCG TCG GCA GCG TCA GAT GTG TAT AAG AGA CAG [3–6‐mer Ns] – [515f] ‐3′; reverse, 5′‐ GTC TCG TGG GCT CGG AGA TGT GTA TAA GAG ACA G [3–6‐mer Ns] ‐ [806r] ‐3′) [13]. The multiplex PCR was performed using KAPA3G Plant PCR kit (KAPA Biosystems), 400 nM of each primer (nexF-N3-N6 tagged 16S and nexR-N3-N6 tagged 16S) and 3 µl of cleaved DNA or 1 µl of DNA in a 15 µl reaction volume. The PCR profile was as follows: initial denaturation at 95 °C for 2 min, then 24 cycles of denaturation at 95 °C for 20 s, annealing at 50 °C for 20 s and extension at 72 °C for 30 s, followed by the final extension step at 72 °C for 1 min (BIO-Rad T100™ Thermal Cycler). In vitro cleavage on 50 nM of PCR amplicon was carried out for specific samples using RNP complex mix-3. Cleaved samples were purified using SeraPure magnetic beads following the recommended Ampure magnetic bead protocol. Second step PCR was performed on all samples using the same PCR kit with Fusion primers carrying Illumina indices using 400 nM of each primer and 1.8 µl of 1st step PCR product or 5 µl of purified cleaved 1st step PCR product in an 18 µl reaction volume. The PCR profile was as follows: initial denaturation at 95 °C for 2 min, then 8 cycles of denaturation at 95 °C for 20 s, annealing at 50 °C for 20 s, and extension at 72 °C for 30 s, followed by the final extension step at 72 °C for 2 min (BIO-Rad T100™ Thermal Cycler). Successful amplification of the target region was checked for all PCR samples by separating the PCR product on a 2% agarose gel. Then the PCR amplicons of the samples underwent a purification/equalisation process using SeraPure magnetic beads following the recommended Ampure magnetic bead protocol. Finally, PCR products were eluted in 15 µl of 0.1X TE buffer (10 mM Tris and 1 mM EDTA at pH 8.0) and 2 µl of each PCR product was used for a single pool (1 pool per plate of 96 samples). Each pool was analysed on the Automated Bioanalysis System LabChip® GX Touch HT (Perkin-Elmer), concentration was estimated by Qubit® 2.0. Fluorometer (Invitrogen). The 4 nM final pooled library including all the samples from the experiments was processed on an Illumina MiSeq sequencer with 250 × 2 paired-end mode at the Genomics Facility at the University of Auckland.

The raw binary base call (BCL) data were processed as described in Larrouy et al. [14] and claident databases; prokaryota_16S_genus and prokaryota_16S_species were used. Metataxa2 was used to determine the origin of sequences (bacteria, chloroplast, or mitochondria) in 16S rRNA dataset [15]. Number of sequences from each origin in each sample was calculated using Python Pandas (v1.3.5) [16] and tabulated the package on Linux. Bioinformatic analyses were performed on the NeSI HPC environment (www.nesi.org.nz). The R programming environment with used to calculate the ratio of (bacterial-reads/total-reads) for each sample. Pairwise comparisons using a parametric *t*-test or non-parametric Wilcoxon-test method (depending on the distribution) were performed between control and non-control groups. These ratios were plotted using the Ggplot2 R package [17].

### Testing Cas-16S-seq Method Implemented for *L. scoparium*

Comparison of the Cas-16S-seq method to the standard 16S rRNA amplicon sequencing (16S-seq) was performed on a total of 262 DNA samples. These DNA represented 224 samples from Larrouy et al. [14] corresponding to five floral stages (from immature bud to spent flower) and seeds from three *L. scoparium* ‘Crimson Glory’ plants, one *L. scoparium* ‘EC47’ plant and one *L. scoparium* ‘N3Z7’ plant. In addition, 38 leaf samples from these five plants were collected. This sample set allowed the extension of this method to other *L. scoparium* tissues and genotypes to be determined, the method was performed on *L. scoparium* ‘Crimson Glory’ leaf samples and *L. scoparium* ‘EC47’ and ‘N3Z7’ floral samples.

For the 16S rRNA amplicon sequencing, bacterial communities were characterised from uncleaved DNA using the V4 region of the bacterial 16S rRNA gene as described previously in this paper. The 4-nM final pooled library including all the samples from the experiments was processed on an Illumina MiSeq sequencer with 250 × 2 paired-end mode at the Genomics Facility at the University of Auckland.

For the Cas-16S rRNA amplicon sequencing, in vitro cleavage was performed at 37 °C for 2 h in a 20 µl reaction, 2 µl of RNP (mix-10 = all gRNAs from Table 1), 30 ng of DNA substrate and 2 µl of 10X Cas9 Nuclease Reaction Buffer. The reactions were treated with 2 µl of Proteinase K (20 mg/ml) at 56 °C for 10 min to release the DNA substrate from the Cas9 endonuclease and then purified with SeraPure magnetic beads following the recommended Ampure magnetic bead protocol. The first step PCR was performed as described previously using 3 µl of cleaved DNA as template. Second step PCR, clean-up, and sequencing were performed as described previously.

The same bioinformatic pipeline was used for 16S-seq and Cas-16S-seq methods as described previously. Pairwise comparisons using non-parametric Wilcoxon-test method were performed between 16S-seq and Cas-16S-seq methods.

## Results

### Design and Implementation of Cas-16S-seq Method for *L. scoparium*

**Fig. 1:**
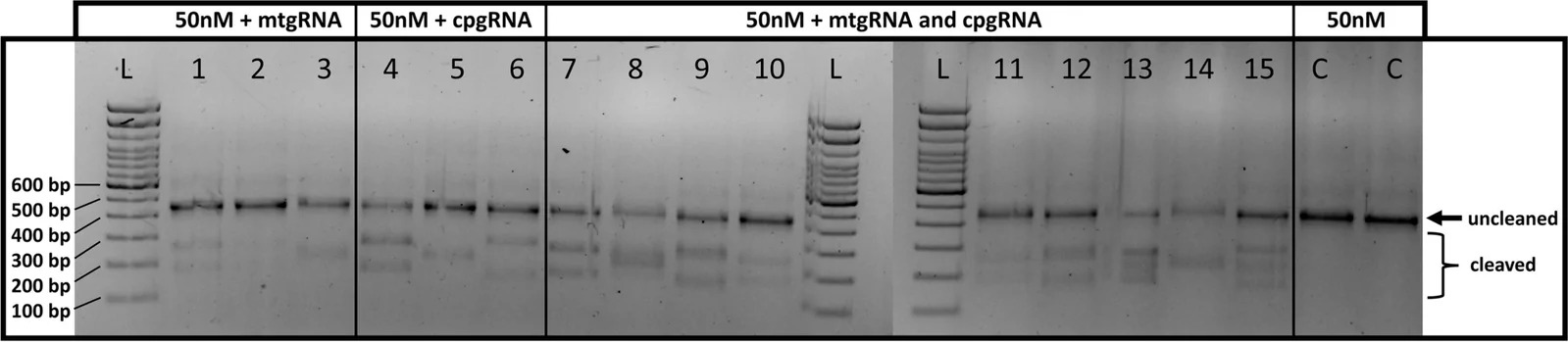
In vitro cleavage using RNP complex on V4-16S rRNA gene amplicons. gRNA showed cleavage efficiency of *Leptospermum scoparium* mitochondrial and chloroplast sequences. Six gRNAs (mt-88, mt-91, mt-107, cp-81, cp-131, and cp-189) were used alone (lanes 1 to 6) or mixed (lanes 7 to 10 and 11 to 15) to evaluate their cleavage performance. Lane 1 is mt-88, lane 2 is mt-91, lane 3 is mt-107, lane 4 is cp-81, lane 5 is cp-131, lane 6 is cp-189, lane 7 is Mix-1 (= mt-88 + cp-81), lane 8 is Mix-2 (= mt-88 + cp-131), lane 9 is Mix-3 (= mt-88 + cp-189), lane 10 is Mix-4 (= mt-91 + cp-81), lane 11 is Mix-5 (= mt-91 + cp-131), lane 12 is Mix-6 (= mt-91 + cp-189), lane 13 is Mix-7 (= mt-107 + cp-81), lane 14 is Mix-8 (= mt-107 + cp-131), and lane 15 is Mix-9 (= mt-107 + cp189). Control samples (lanes C) underwent the same cleavage step without RNP complex

To examine the cleavage efficiency of the Cas9 nuclease and gRNAs, the cleavage of V4-16S rRNA gene amplicons obtained from *L. scoparium* ‘Crimson Glory’ flower samples using the three mt-gRNAs and the three cp-gRNAs individually or mixed were visualised on an agarose gel. The size of uncleaved amplicons was ~ 480 bp and the expected size of cleaved amplicons was between ~ 175 and 305 depending on the gRNA tested (expected size of cleaved amplicon for each gRNA alone or mixed are recorded in Table S1). All gRNAs individually or mixed showed a cleavage (Fig. 1), with the uncleaved control amplicon appearing darker demonstrating a higher concentration of uncleaved amplicon in the control treatment compared to all gRNA treatment. However, the efficiency could not be calculated due to size similarity between mitochondrial, chloroplast, and bacterial sequences.

**Fig. 2:**
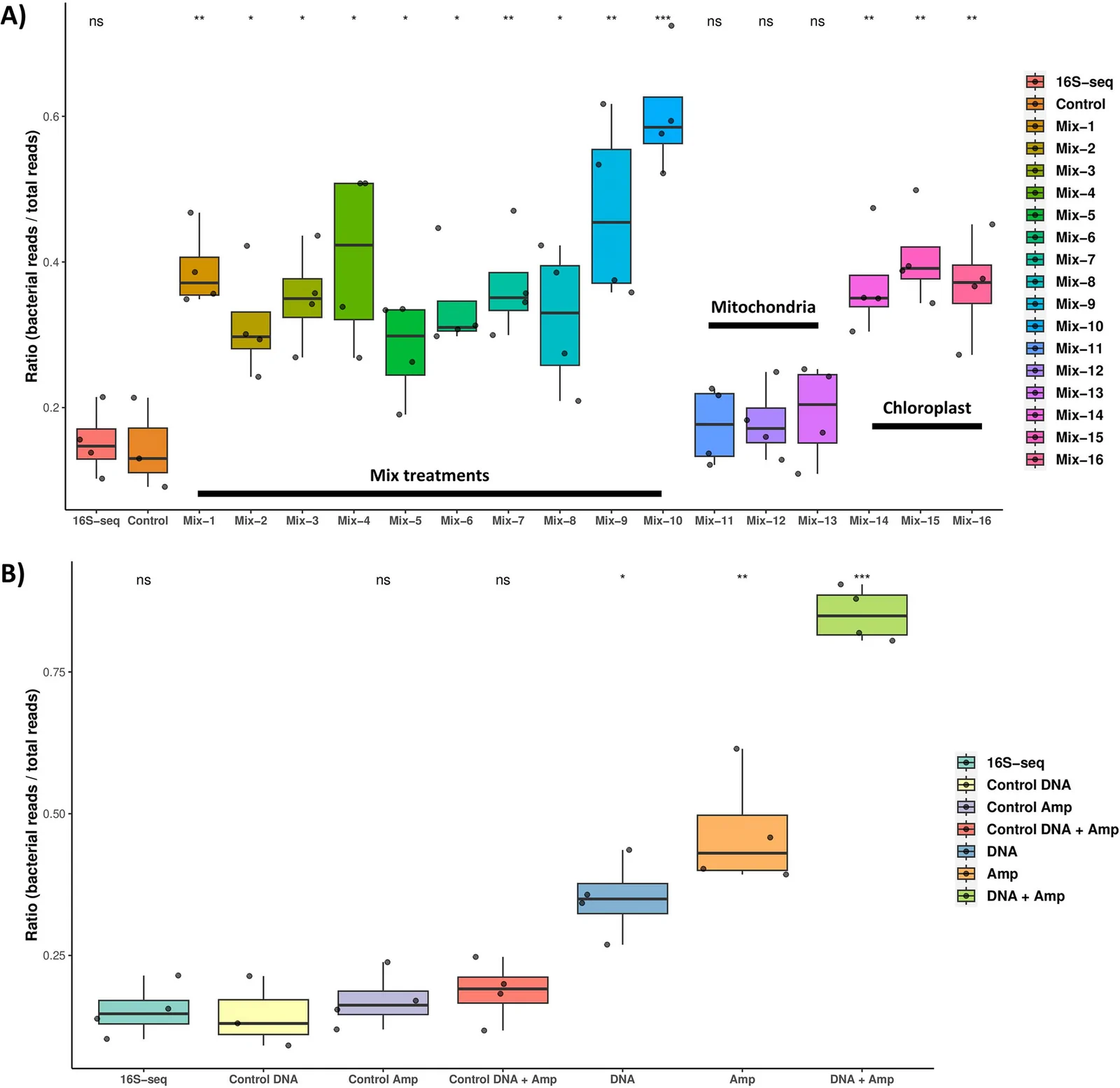
Implementation of the Cas-16S-seq method on *Leptospermum scoparium* ‘Crimson Glory’ floral DNA. **A** Evaluation of the gRNA combinations on eliminating plastid and mitochondrial sequences. Box plot representing the ratio bacterial reads on total reads for each sample. Samples are grouped by condition, 16S-seq = library preparation without the use of CRIPSR-Cas9. Control = samples underwent the same cleavage step without RNP complex, Mix-1 = mt-88 + cp-81, Mix-2 = mt-88 + cp-131, Mix-3 = mt-88 + cp-189, Mix-4 = mt-91 + cp-81, Mix-5 = mt-91 + cp-131, Mix-6 = mt-91 + cp-189, Mix-7 = mt-107 + cp-81, Mix-8 = mt-107 + cp-131, Mix-9 = mt-107 + cp-189, Mix-10 = all gRNAs, Mix-11 = mt-88 + mt-91, Mix-12 = mt-88 + mt-107, Mix-13 = mt-107 + mt-91, Mix-14 = cp-81 + cp-131, Mix-15 = cp-81 + cp-189, and Mix-16 = cp-131 + cp-189. *T*-test results using Control samples as reference are shown as non-significant (ns) or significant (*p ≤ 0.05; **p ≤ 0.01; ***p ≤ 0.001). **B** Evaluation of the enzymatic cleavage at the different steps of library preparation on eliminating plastid and mitochondrial sequences. Box plot representing the ratio bacterial reads on total reads for each sample. Samples are grouped by condition, 16S-seq = library preparation without the use of CRIPSR-Cas9, Control DNA = samples underwent the same cleavage at DNA step without RNP complex, Control Amp = samples underwent the same cleavage at 1st step PCR amplicon step without RNP complex, Control DNA + Amp = samples underwent the same cleavage at DNA and 1st step PCR amplicon step without RNP complex, DNA = library preparation with cleavage step on DNA, Amp = library preparation with cleavage step on 1st step PCR amplicon, DNA + Amp = library preparation with cleavage step on DNA and 1st step PCR amplicon. In vitro cleavage used RNP including mix-3 (gRNAs mt-88 and cp189). *T*-test results using Control-DNA samples as reference are shown as non-significant (ns) or significant (*p ≤ 0.05; **p ≤ 0.01; ***p ≤ 0.001)

To explore the performance of the gRNAs in the Cas-16S-seq method to remove host contamination in V4-16S amplicons, the ratio (bacterial reads/total reads) was calculated for each sample (Fig. 2A). The results showed that there was no significant difference (*p* = 0.86) in the ratio of the 16S-seq method (library preparation without CRISPR-Cas9 tool) and the control method (samples that underwent the same cleavage step without RNP complex). All samples treated with RNP mixes (Table 2) containing gRNAs targeting chloroplast and mitochondrial sequences (mix-1 to mix-10) increased their ratio compared to the control (all *p* < 0.05). No significant differences were observed for samples treated with RNP containing gRNA targeting only the mitochondrial sequences (mix-11 to mix-13) (all *p* > 0.05). Samples treated with RNP containing gRNA targeting the chloroplast sequences showed significant increases in their bacterial:total reads ratio compared to the control conditions (mix-14 to mix-16) (all *p* < 0.05). The most efficient gRNA combination was mix-10 (= all gRNAs in RNP complex) with a significant increase in bacterial:total reads ratio from 0.13 (control condition) to 0.59 (mix-10 condition, *p* = 4.5e − 4). These findings illustrated a substantial 46% enhancement in bacterial sequence presence within DNA samples cleaved utilizing the RNP complex containing all gRNAs.

To optimise the Cas-16S-seq method, in vitro cleavage to remove host contamination was tested at different steps of the library preparation protocol; DNA, 1st step PCR amplicon (Amp) or both (DNA + Amp) (Fig. 2B). No significant difference was observed in the bacterial:total reads ratio under the following conditions: 16S-seq (library preparation without the use of CRIPSR-Cas9), control DNA samples (no RNP complex at the cleavage step), control Amp (no RNP complex at the cleavage step) and control DNA + Amp (no RNP complex at both cleavage steps) (all *p* =  ≥ 0.38). Thus, all conditions were compared to control DNA. Significant differences were found between DNA, Amp, DNA + Amp, and the control DNA condition (*p* = 0.01, 3.8e − 3 and 1.5e − 4, respectively). A significant increase in the ratio from 0.13 (control DNA condition) to 0.35 (DNA condition) corresponded to an increase of 22% of bacterial reads. A significant increase in the ratio from 0.13 (control DNA condition) to 0.43 (Amp condition), corresponded to an increase of 30% of bacterial reads. A significant increase in the ratio from 0.13 (control DNA condition) to 0.85 (DNA + Amp condition) corresponded to an average increase of 72% of bacterial reads. No significant difference was found between DNA and amplicon conditions, whereas the DNA + Amp condition showed a significantly higher ratio than the DNA and Amp conditions (*p* = 4.5e − 5 and 1.9e − 3, respectively).

**Fig. 3:**
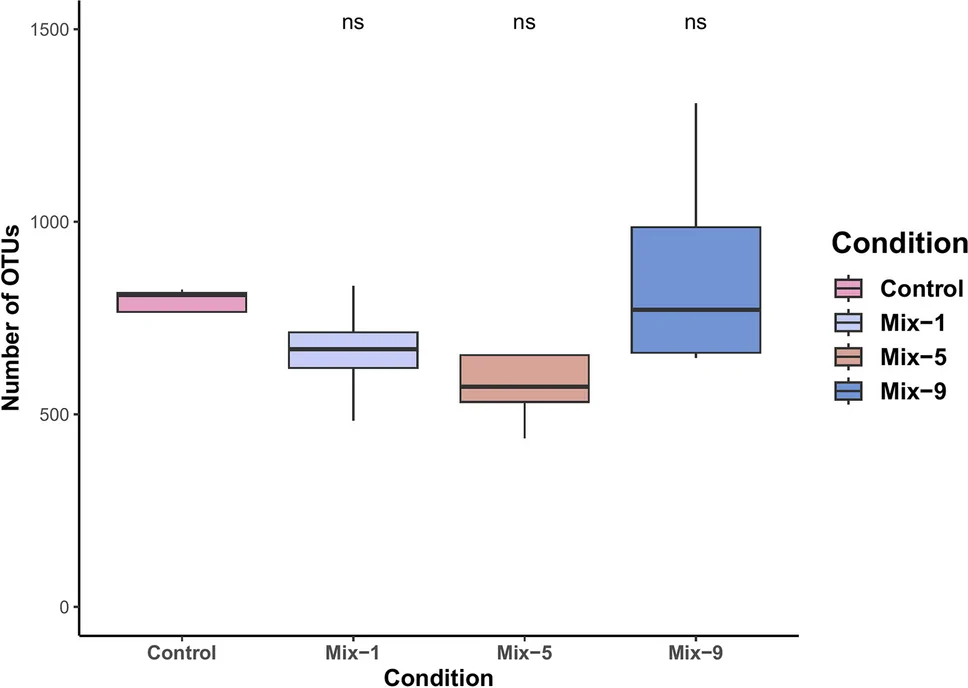
Number of bacterial OTUs retrieved in four soil samples using 16S-seq and Cas-16S-seq methods. Evaluation of the gRNAs on introducing bias in bacterial OTUs recovery from soil samples. Box plot representing the number of OTUs of four soil samples per condition. Control = samples underwent the same cleavage step without RNP complex, Mix-1 = mt-88 + cp-81, Mix-5 = mt-91 + cp-131, and Mix-9 = mt-107 + cp-189. Wilcoxon-test results using Control samples as reference are shown as non-significant (ns) or significant (*p ≤ 0.05; **p ≤ 0.01; ***p ≤ 0.001)

To define off-target bias from gRNAs, Cas-16S-seq using gRNAs mixes 1, 5, and 9 was tested on four soil samples. In all soil samples, the percentage of bacterial read was 100%. No significant difference in the number of Operational taxonomic units (OTUs) was observed between 16S-seq and Cas-16S-seq for the different gRNAs mixes 1, 5, and 9 (all* p* =  ≥ 0.2, Fig. 3).

### Comparison 16S-seq vs Cas-16S-seq Method on *L. scoparium* Tissue Types

To explore the performance of the Cas-16S-seq method to remove host contamination in *L. scoparium* V4-16S amplicons and the representation of bacterial amplicons, 16S-seq vs Cas-16S-seq methods were compared on a large number of floral DNA samples (including five floral stages), seed and leaf DNA from five *L. scoparium* plants representing three genotypes. The ratio (bacterial reads/total reads) was calculated for each sample and the average ratios (bacterial reads/total reads, chloroplast reads/total reads, and mitochondrial reads/total reads) were calculated from all samples for the 16S-seq or Cas-16S-seq methods (Table 3).

**Table 3:** Dataset comparison between 16S-seq and Cas-16S-seq methods. The table includes the number of samples retrieved from each experiment, total number of reads (= sequences), number of bacterial reads and the ratio of bacterial reads of the total reads, number of chloroplast reads and the ratio of chloroplast reads of the total reads, number of mitochondrial reads and the ratio of mitochondria reads of the total reads, number of eukaryota reads and the ratio eukaryota reads on total of reads, number of uncertain reads (where no origin was determined by Metataxa2) and the ratio of uncertain reads on total of reads

	*16S-seq*	*16S-seq ratio (reads/total reads)*	*Cas-16S-seq*	*Cas-16S-seq ratio (reads/total reads)*
*Number of samples*	258		262	
*Total reads*	1,909,526	1.00000	1,981,584	1.00000
*Bacterial (include Archaea) reads*	171,585	0.08986	589,321	0.29740
*Chloroplast reads*	1,261,014	0.66038	908,258	0.45835
*Mitochondria reads*	461,820	0.24185	403,194	0.20347
*Eukaryota reads*	15,037	0.00787	3014	0.00152
*Uncertain reads*	70	0.00004	77,797	0.03926

Using the 16S-seq method, a total of 1,909,526 sequences were retrieved (258 samples) (Table 3), 9% of sequences were from bacterial origin, 66% of sequences were from chloroplast origin, and 24% sequences were from mitochondrial origin. Using the Cas-16S-seq method, 1,981,584 sequences were retrieved, 30% of the sequences were from bacterial origin, and 45% of sequences were from chloroplast origin and 20% of the sequences were from mitochondrial origin. Cas-16S-seq method increased the proportion of bacterial sequences by 20% in the dataset compared to 16S-seq method. In *L. scoparium* ‘Crimson Glory’ floral samples (immature bud to spent flower), the Cas-16S-seq method increased the proportion of bacterial sequences in the dataset by 30% compared to 16S-seq method, by 22% for leaf samples and by 30% for seed samples. In the two other genotypes of *L. scoparium*, the Cas-16S-seq method increased the proportion of bacterial sequences in the dataset by 5% compared to 16S-seq method, by 12% for leaf samples and by 2.5% for seed samples (Table 4).

**Table 4:** Statistical comparison of dataset retrieved from 16S-seq and Cas-16S-seq methods. The table includes the number of samples in each group (16S-seq and Cas-16S-seq), the ratio of bacterial reads out of the total reads for each group, and the *p*-value from the Wilcoxon test performed on the bacterial ratios between each method for each group

	*16S-seq ratio bacterial reads*	*Cas-16S-seq ratio bacterial reads*	*P-value Wilcoxon-test between ratio of 16S and Cas-16S*
*Crimson glory all floral sample (n* = *109, n* = *113)*	0.16	0.48	4.60E − 16
*Crimson glory leaf samples (n* = *24, n* = *23)*	0.04	0.26	6.40E − 06
*Crimson glory seed samples (n* = *23, n* = *24)*	0.12	0.42	7.60E − 08
*EC/N3 all floral sample (n* = *71, n* = *71)*	0.02	0.07	2.00E − 07
*EC/N3 leaf samples (n* = *16, n* = *16)*	0.05	0.17	3.70E − 05
*EC/N3 seed samples (n* = *16, n* = *16)*	0.005	0.03	0.013

All samples from Cas-16S-seq had optimum sequencing coverage whereas only three samples from the 16S-seq method had optimum coverage (100/100).

**Fig. 4:**
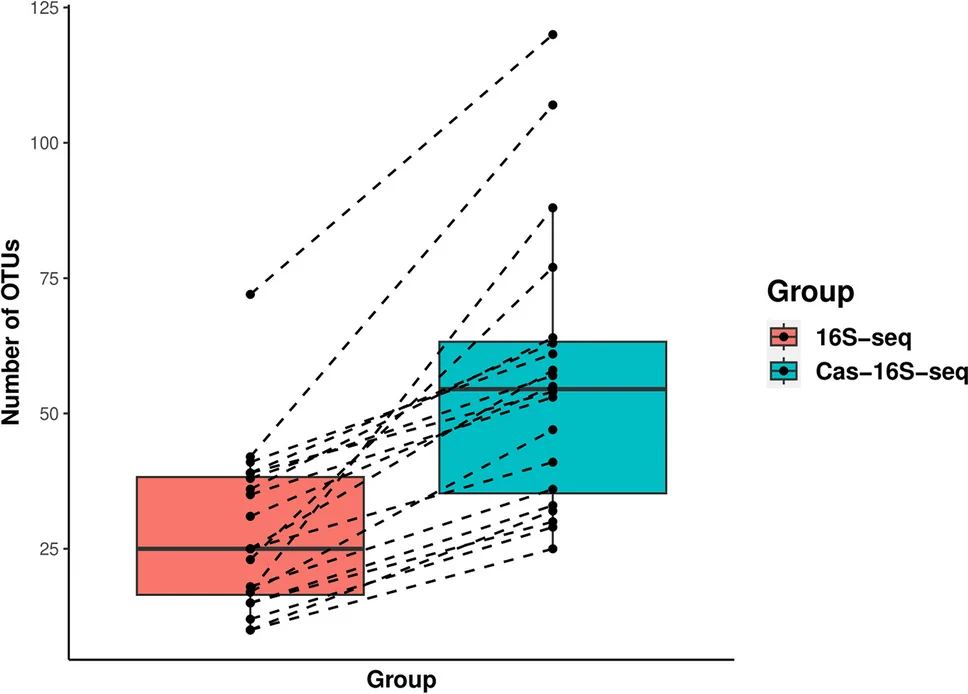
Number of OTUs from *L. scoparium* samples processed with 16S-seq and Cas-16S-seq. This represents a subset of samples (*n* = 20) from the comparison of the Cas-16S-seq method to the standard 16S rRNA amplicon sequencing (16S-seq) performed on a total of 262 DNA samples. The lines connect the sample that was process with both method for direct comparison of the number of OTUs

Sequencing datasets underwent clean-up steps (removing control samples, contaminant sequences, low abundance OTUs, and samples with low number of reads). Clean datasets for both methods were compared (Table 5). Cas-16S-seq dataset was composed of three times more sequences and close to three times more OTUs than the 16S-seq dataset. The increased in number of OTUs in sample processed using Cas-16S-seq compared to 16S-seq is shown in Fig. 4 on a subset of samples. Finally, in the Cas-16S-seq dataset approximately two times as many phyla, over three times more families, and four times more genera were retrieved compared to the 16S-seq dataset.

**Table 5:** Cleaned dataset comparison between 16S-seq and Cas-16S-seq samples. Through clean-up steps, plants read, controls, sample < 2 reads, OTUs < 2 reads were removed for 16S-seq dataset and plants reads, controls, sample < 10 reads, OTUs < 10 and outlier samples reads were removed for Cas-16S-seq dataset. A total of 253 samples for 16S-seq and 235 samples for Cas-16S-seq were retrieved after clean-up steps

	*16S-seq*	*Cas-16S-seq*
*Number of sequences*	171,579	580,275
*Average number of reads*	678	2358
*Number of OTUs*	178	495
*Number of Phyla*	10	19
*Number of families*	41	140
*Number of genera*	49	188

## Discussion

Understanding host-microbe interactions *in planta* is an expanding area of research and 16S rRNA sequencing is a powerful and common method to study the microbiota. However, the co-amplification of mitochondrial and plastid 16S rRNA genes by universal primers impairs the sensitivity and performance of 16S rRNA sequencing. Song and Xie [11] developed an effective method, Cas-16S-seq, to remove host contamination, when profiling the microbiota of rice samples, by engineering RNA-programmable Cas9 nuclease in 16S-seq. Their method was highly efficient without introducing bias for this well-studied domesticated plant. Thus, in this study, we tested the Cas-16S-seq method on the bacterial communities associated with foliage and flowers of a wild plant species, *L. scoparium*. This is the first study to independently evaluate the Cas-16S-seq method on a wild species for which there is limited genomic information.

This study demonstrated that the Cas-16S-seq method for *L. scoparium* was efficient in removing host contamination in V4-16S amplicons. An increase of 46% in bacterial sequences was found using all gRNAs in the same reaction and an increase of 72% in bacterial sequences was obtained when using Cas9-gRNAs complex, cleaving plant sequences, at two different steps of the library preparation (DNA and 1st step PCR amplicon) for the same sample. The number of OTUs retrieved from soil samples was consistent when using the different methods (Cas-16S-seq and 16S-seq) indicating that the Cas-16S-seq method does not return a reduced sequencing profile. Indeed, a similar number of OTUs in soil samples processed with both the Cas-16S-seq and the 16S-sequencing method indicated that the gRNAs for *L. scoparium* did not target bacterial 16S rRNA genes. This was also demonstrated by Song and Xie for their work on rice.

Song and Xie [11] cleaved amplicons from the 1st step PCR. Amplification of specific sequence targets relies on the fractions that those sequences present within the total DNA template. Because relative amplification efficiency is nonlinear, low-proportion taxa in a community can be under-represented during PCR-based surveys, and a large number of sequences might need to be processed to detect rare bacterial taxa [18]. Indeed, using Cas9-gRNAs complex to cleave plant DNA allows the amplification of bacterial sequence in the first PCR, whereas the cleavage on 1st step PCR amplicons allows the amplification, at the 2nd step PCR, of only the bacterial product amplified in the first PCR. Therefore, in the method implemented for *L. scoparium*, the cleavage was performed on genomic DNA to maximise the detection of rare OTUs.

*L. scoparium* Cas-16S-seq efficiency was significantly higher by cleaving plant sequences of the same sample twice, at two steps of the library preparation. These allow both the targeting of plant organelle DNA within the total DNA template first, as well as residual amplicons generated from organelle DNA missed in the first cleavage. Increasing the number of steps and the resulting manual handling of samples during library preparation can potentially increase sample contamination [19]. Thus, the comparison of both methods on a large number of samples was performed using a Cas9-gRNAs complex on genomic DNA for Cas-16S-seq.

This study demonstrated that Cas-16S-seq method implemented for *L. scoparium* outperformed the 16S-seq method for the recovery of bacterial sequences. Indeed, on multiple samples, including other genotypes of *L. scoparium* and other tissue types such as leaves and seeds, the *L. scoparium* Cas-16S-seq method increased the number of bacterial sequences by 20% overall, with 48% of the sequences obtained from all floral *L. scoparium* ‘Crimson Glory’ samples bacterial in origin. The Cas-16S-seq method increased the *L. scoparium* ‘Crimson Glory’ sequencing coverage (sequencing depth) to a level sufficient for *L. scoparium* microbiota profiling and increased (almost triple compared to 16S-seq method) number of OTUs, phyla, families, and genera retrieved. However, it is important to note that even though the number of plant sequences was greatly reduced and the community profiling was improved significantly using this method, a high percentage of *L. scoparium* sequences was still retrieved. The use of Cas9-gRNAs complex twice during the library preparation, DNA and 1st step amplicon, would have allowed a higher bacterial sequence recovery and is recommended for future work.

The efficiency of the Cas-16S-seq is not the only advantage of this method. Song and Xie [11] emphasised other advantages such as the low cost and the simplicity of the method relative to other enrichment processes. In this study, we did not compare this method to another available; however, the design of gRNAs targeting *L. scoparium* sequences, using the bioinformatic pipeline and testing to determine if the design gRNAs can cleave the target DNA, was easy and quick to perform. The gRNA design can be performed using free online tools (e.g. IDT, CRISPR-P), the gRNAs and Cas9 enzyme are commercially available or can be readily prepared in the laboratory, and the method (cleavage reaction using Cas9-gRNAs complex) is not time-consuming, easy to apply, and can be integrated into current 16S rRNA gene-based amplicon sequencing workflows. Even if the design of gRNA for Cas-16S-seq is more challenging than other CRISPR/Cas9 applications as the gRNA is required to discriminate one target from numerous homologs, Song and Xie [11] designed a sophisticated pipeline to identify unwanted bacterial targets of the designed gRNAs that is easily to apply to other target sequences. However, in less studied plants, such as *L. scoparium*, the genome is not always available. Mitochondrial and chloroplast sequences will therefore need to be obtained prior to designing the gRNAs, increasing the cost and time in the design which is a potential limitation of the method’s application to less described or wild species.

Cas9/gRNA can efficiently cleave DNA that perfectly matches gRNA but also cleave partially matched off-targets with reduced activity. Due to the *L. scoparium* genome being unavailable at the time, the search for gRNAs off-target against the *L. scoparium* full genome was not performed in silico. *L. scoparium* designed gRNAs could have potential off-target in other parts of the *L. scoparium* genome when performing the cleavage at the DNA level. Therefore, we tested the efficiency and specificity of the different selected gRNAs. Since the design of *L. scoparium* gRNA in the Cas-16S-seq method was based on known 16S rRNA genes, potential off-targets in uncharacterised bacteria cannot be fully excluded. The designed gRNAs for *L. scoparium* ‘Crimson Glory’ were highly specific, as they were not as efficient on the other genotypes of *L. scoparium*, due to difference in 16S sequences, demonstrating the very low risk of off-target sequence amplification. When studying different genotypes of different plant species, designing, and testing the gRNAs for each of the genotypes and/or species could increase the time and cost of the method. Sequencing the 16S rRNA gene of different plant genotypes before creating the gRNAs would overcome this limitation. The gRNAs mt-88 and cp-189 were tested on a small set of *Metrosideros excelsa* and *Lophomyrtus bullata* leaf and flower samples. Their efficiency on the samples tested was low. However, one of the gRNA that was designed in this study 5′- TGAAAGTCGCCAAAAAGTGG-3′ (mt-91) is identical to mt-gRNA662 + designed by Song and Xie [11] targeting *Oryza sativa L. ssp geng* cultivar Nipponbare mitochondrial 16S rRNA gene. This shows that the same gRNA could be used for different plant species, and eventually, the uptake of this method could create a pool of validated and published gRNAs. Finally, as for any other method to mitigate host contamination in 16S rRNA sequencing, confirming the efficiency of the method can only be done by library preparation and NGS sequencing and therefore has an often-significant cost.

After comprehensive comparisons of Cas-16S-seq and regular 16S-seq data on multiple *L. scoparium* flower, leaf, and seed samples, we demonstrated that Cas-16S-seq reduced the host 16S rRNA fraction but did not introduce bias to the microbiota profiles. In conclusion, Cas-16S-seq method is an efficient and promising method to overcome the major issue of host contamination in bacterial profiling microbiota associated with plants. Cas-16S-seq is a major advancement in plant microbiota research and will likely become a prominent method in this field.

## Supplementary Information

Below is the link to the electronic supplementary material.

Supplementary file1 (DOCX 17 kb)

## Data Availability

Permission from stakeholders representing the appropriate indigenous Māori iwi was obtained for using the plant material in this study. The data supporting the findings of this study is available upon request from the corresponding author, subject to consent from the Māori iwi.
