# Supplementary material for: Improvement in Microbiota Recovery Using Cas-9 Digestion of Mānuka Plastid and Mitochondrial DNA
Source: Microb Ecol. 2024 Oct 9;87:124. doi: 10.1007/s00248-024-02436-6 (PMC11461681; doi:10.1007/s00248-024-02436-6)
Supplement: Supplementary file 1 [file 248_2024_2436_MOESM1_ESM.docx]

Supplementary data

**Table S1:** Expected size of cleaved amplicon (2nd step PCR ~ 480 bp) for the six gRNA and the nine mixes.

| **gRNA_ID** | **Mix number** | **Cleaved product sizes of the 2nd step amplicon ~ 480 bp** |
| --- | --- | --- |
| **cp-131** |  | ~245,~235 |
| **cp-189** |  | ~305, ~175 |
| **cp-81** |  | ~285, ~195 |
| **mt-88** |  | ~275, ~205 |
| **mt-91** |  | ~275, ~205 |
| **mt-107** |  | ~255, ~225 |
| **mt-88 and cp-81** | Mix-1 | ~275,~285,~205,~195 |
| **mt-88 and cp-131** | Mix-2 | ~275,~245,~205,~235 |
| **mt-88 and cp-189** | Mix-3 | ~275,~305,~205,~175 |
| **mt-91 and cp-81** | Mix-4 | ~275,~285,~205,~195 |
| **mt-91 and cp-131** | Mix-5 | ~275,~245,~205,~235 |
| **mt-91 and cp-189** | Mix-6 | ~275,~305,~205,~175 |
| **mt-107 and cp-81** | Mix-7 | ~255,~285,~225,~195 |
| **mt-107 and cp-131** | Mix-8 | ~255,~245,~225,~235 |
| **mt-107 and cp-189** | Mix-9 | ~255,~305,~225,~175 |
